# Genetic variability of human adenovirus type 7 circulating in mainland China

**DOI:** 10.1371/journal.pone.0232092

**Published:** 2020-04-30

**Authors:** Ru Cai, Naiying Mao, Jingjing Dai, Xingyu Xiang, Jing Xu, Yingwei Ma, Zhong Li, Guangyue Han, Deshan Yu, Jie Yin, Aili Cui, Yan Zhang, Hong Li, Pengbo Yu, Luyuan Guan, Yuling Tian, Liwei Sun, Yan Li, Yamei Wei, Zhen Zhu, Wenbo Xu

**Affiliations:** 1 Medical School, Anhui University of Science and Technology, Huainan city, Anhui province, People’s Republic of China; 2 NHC Key Laboratory of Medical Virology Ministry of Health, National Institute for Viral Disease Control and Prevention, Chinese Center for Disease Control and Prevention, Beijing, People’s Republic of China; 3 Department of Medical Laboratory, the Affiliated Huai’an No. 1 People’s Hospital of Nanjing Medical University, Huai’an city, Jiangsu province, People’s Republic of China; 4 Hunan Provincial Center for Disease Control and Prevention, Changsha city, Hunan province, People’s Republic of China; 5 Shaanxi Provincial Center for Disease Control and Prevention, Xi’an city, Shaanxi province, People’s Republic of China; 6 Changchun Children’s Hospital, Changchun city, Jilin province, People’s Republic of China; 7 Shandong Provincial Center for Disease Control and Prevention, Jinan city, Shandong province, People’s Republic of China; 8 Hebei Provincial Center for Disease Control and Prevention, Shijiazhuang city, Hebei province, People’s Republic of China; 9 Gansu Provincial Center for Disease Control and Prevention, Lanzhou city, Gansu province, People’s Republic of China; 10 Yunnan Provincial Center for Disease Control and Prevention, Kunming city, Yunnan province, People’s Republic of China; 11 The Affiliated Hospital of Southwest Medical University, Luzhou city, Sichuan province, People’s Republic of China; University of Nantes, FRANCE

## Abstract

Human adenovirus (HAdV-7) is a highly contagious pathogen that causes severe respiratory illnesses. However, the epidemic patterns and genetic variability of HAdV-7 circulating in mainland China have not been well elucidated. In this study, we used Chinese HAdV sentinel surveillance data obtained from 2012–2015 to investigate the clinical features of 122 HAdV-7-positive cases and performed amplification and sequence determination of three capsid genes (penton base, hexon, and fiber) from 69 isolated viruses covering from seven provinces of China. Additionally, we compared with data from representative sequences of 21 strains covering seven more provinces in China and 32 international HAdV-7 strains obtained from GenBank database to determine the phylogenetic, sequence variations, and molecular evolution of HAdV-7. The results indicated that HAdV-7 infection occurred throughout the year, and a high proportion of severe cases (27 cases, 22.1%) exhibited infantile pneumonia. Moreover, phylogenetic analysis showed that all HAdV-7 strains could be divided into two major evolutionary branches, including subtype 1 and subtype 2, and subtype 3 was also formed according to analysis of the penton base gene. Subtypes 1 and 2 co-circulated in China before 2008, and HAdV-7 strains currently circulating in China belonged to subtype 2, which was also the predominant strain circulating worldwide in recent years. Further sequence variation analysis indicated that three genes of HAdV-7 were relatively stable across time and geographic space, particularly for viruses within subtypes, which shared almost the same variation sites. Owing to continuous outbreaks caused by HAdV-7, resulting in increased illness severity and fatality rates in China, the establishment of a national HAdV surveillance system is urgently needed for the development of effective preventive and infection-control interventions for adenovirus respiratory infections in China.

## Introduction

Human adenoviruses (HAdVs), which belong to the genus *Mastadenovirus* in the family *Adenoviridae*, are nonenveloped, double-stranded linear DNA viruses with an icosahedral capsid [1]. Hexon, fiber, and penton base are the three major capsid proteins of HAdV and are usually used for genotype identification [1]. Based on their biological properties and genomic sequence features, HAdVs can be classified into seven species (A–G) and at least 103 genotypes, which are assigned by the Human Adenovirus Working Group (July 2019 update, http://hadvwg.gmu.edu/).

HAdV is a highly contagious pathogen that can cause a variety of diseases owing to variations in tissue tropism and virulence. Several genotypes of HAdV, including species B (HAdV-3, -7, -11, -14, -16, -21, -50, -55), species C (HAdV-1, -2, -5, -6), and species E (HAdV-4), have tropism for the respiratory tract and are commonly associated with adenoviral respiratory infections [2, 3]. Among these genotypes, HAdV-3 and -7 are the most common causative agents worldwide [4–7]; HAdV-7 is the most pathogenic and can cause more severe respiratory illness and higher fatality rates than other HAdV types [8]. Outbreaks of HAdV-7 have been reported at military training bases, school clusters, and communities around the world [2, 9–14].

Although China has not yet established a complete adenovirus surveillance system, HAdV sentinel surveillance was recently integrated into the febrile respiratory syndrome (FRS) surveillance program supported by the Key Technologies R&D Program of the National Ministry of Science during 2012–2015. With the HAdV-7 strains obtained through this program, we aimed to study the epidemic patterns, genetic variability, and molecular evolution of HAdV-7 based on the three major capsid proteins. This information will be essential for the development of effective strategies for the prevention and control of adenoviral respiratory infections in China.

## Materials and methods

### Ethical statement

This study was approved by the second session of the Ethics Review Committee of the National Institute for Viral Disease Control and Prevention (IVDC) at China Centers for Disease Control and Prevention (CDC). All participants or legal guardians involved in this study provided written informed consent to have data/samples from their medical records used in research. All methods were performed in accordance with the relevant guidelines and regulations.

### Sources of HAdV-7 virus strains

According to the FRS program, surveillance provinces collected respiratory specimens, including nasopharyngeal swabs, bronchoalveolar lavage fluid, and sputum, from outpatients and hospitalized patients meeting the case definition [15]. The samples were then transported to the IVDC of China CDC under the cold chain for further identification. During 2012–2015, 5419 patients were enrolled, and their clinical specimens were collected. Pathogenic screening of common respiratory viruses was performed, and the baseline HAdV-positive rate was 3.7% (201 cases). Among these positive cases, 122 specimens were positive for HAdV-7 (60.7%), and no other respiratory viruses were detected. After three passages in HEp-2 human larynx epidermoid carcinoma cells, only 69 strains covering eastern (Shaanxi/8/2012–2013, Gansu/15/2015, Yunnan province/3/2013), central (Hunan/32/2012–2014, Jilin/5/2013–2014), and western regions (Hebei/2/2013–2014, Shandong/4/2013–2014) of China were obtained. Information for 122 HAdV-7 infection cases in this study is listed in S1 Table, and the 69 HAdV-7 strains are listed in Table 1.

**Table 1:** The list of 69 HAdV-7 strains isolated in China in this study.

Strain Name	Province	Gender	Age	Date of onset	Clinical diagnosis	No. of strains with the identical three-gene- sequences in the same province/Year crossed	Genbank No.		
							Penton base	Hexon	Fiber
Hunan2012- E109	Hunan	Male	6m	2012/5/13	Severe pneumonia	/	MT019924	MT019906	MT019942
Hunan2012- E009	Hunan	Male	16m	2012/1/17	Severe pneumonia	11/2012-2014	MT019925	MT019907	MT019943
Hunan2012- E100	Hunan	Female	8m	2012/4/30	Severe pneumonia	9/2012-2014	MT019926	MT019908	MT019944
Hunan2012-E103	Hunan	Male	12m	2012/5/15	Severe pneumonia	2/2012	MT019927	MT019909	MT019945
Hunan2012- E114	Hunan	Male	6m	2012/6/5	Severe pneumonia	/	MT019928	MT019910	MT019946
Hunan2012- E134	Hunan	Male	3y	2012/7/15	Bronchopneumonia	/	MT019929	MT019911	MT019947
Hunan2013-E006	Hunan	Male	6y	2013/1/6	Bronchopneumonia	/	MT019930	MT019912	MT019948
Hunan2013-E018	Hunan	Male	6m	2013/1/22	Bronchopneumonia	5/2012-2013	MT019931	MT019913	MT019949
Hunan2013-026	Hunan	Male	18m	2013/2/18	Severe pneumonia	/	MT019932	MT019914	MT019950
Shaanxi2012-12011	Shaanxi	Male	18y	2012/3/20	Upper respiratory tract infection	8/2012-2013	MT019933	MT019915	MT019951
Shandong2013-0239	Shandong	Female	2y	2013/1/30	Upper respiratory tract infection	4/2013-2014	MT019934	MT019916	MT019952
Hebei2013-2678	Hebei	Male	23y	2013/2/21	Upper respiratory tract infection	/	MT019935	MT019917	MT019953
Hebei2014-8443	Hebei	Male	25y	2014/3/7	Pneumonia	/	MT019936	MT019918	MT019954
Yunnan2013-0266	Yunnan	Female	5y	2013/7/18	Tonsillitis	3/2013	MT019937	MT019919	MT019955
Jilin2013-120	Jilin	Female	4y	2013/10/11	Bronchopneumonia	/	MT019938	MT019920	MT019956
Jilin2014-277	Jilin	Female	4y	2014/4/16	Bronchopneumonia	3/2014	MT019939	MT019921	MT019957
Jilin2014-282	Jilin	Male	2y	2014/5/17	Bronchopneumonia	/	MT019940	MT019922	MT019958
Gansu2015-089	Gansu	Female	4y	2015/1/20	Bronchopneumonia	15/2015	MT019941	MT019923	MT019959

### Amplification and sequencing of penton base, hexon, and fiber genes

The viral DNA was extracted from 69 HAdV-7 virus strains using a QIAamp DNA Mini Kit (Qiagen, Valencia, CA, USA) according to the manufacturer’s instructions. Previously reported primer pairs of HAdV-7 were used to amplify and sequence the entire penton base (1635 bp), hexon (2805 bp), and fiber genes (978 bp) [3]. As reported previously [3], overlapping polymerase chain reaction (PCR) fragments were amplified with Platinum PCR SuperMix (Invitrogen, Carlsbad, CA, USA), purified with a QIAgel Extraction Kit (Qiagen), and sequenced using classical Sanger sequencing and a 3130 Genetic Analyzer (Life Technologies, Japan). The raw sequence data were edited and spliced using Sequencher software Version 5.0 (Gene Codes Corporation, Ann Arbor, MI, USA). Any ambiguous nucleotide sites were resequenced to obtain high-quality sequences from three major capsid genes for further analysis.

### Datasets

As of September 2019, 371 sequences for complete penton base (80 sequences), hexon (152 sequences), and fiber genes (139 sequences) of HAdV-7 were obtained from GenBank database. Patented sequences, sequences with unknown collection years and geographic information, sequences with consistent strain names, and identical sequences in the same country from the same year were excluded. Therefore, in addition to the sequences of 69 HAdV-7 strains in this study, the final datasets contained 33 representative sequences of HAdV-7 for the penton base gene (China: 18/2007–2018; United States of America [USA]: 13/1954–2017; Russia: 2/2013–2014), 45 representative sequences for the hexon gene (China: 21/2007–2018; USA: 16/1954–2017; Russia: 2/2013–2014; Taiwan, China: 3/2002–2011; Korea: 2/1995–1999; Japan: 1/1995), and 48 representative sequences for the fiber gene (China: 20/2007–2018; USA: 16/1954–2017; Russia: 2/2013–2014; Taiwan, China: 1/2011; Korea: 7/1995–2006; Japan: 2/1992–1995). Representative HAdV-7 strains from GenBank database used in this study are listed in Table 2.

**Table 2:** The list of representative HAdV-7 strains from Genbank database used in this study.

No.	Strain Name in GenBank	Country/Province	Collection Date	Genotype	Accession Number			Lineage		
					P	H	F	P	H	F
1	Gomen	USA	1954	7p	AY594255			1	1	1
2	gz07	CHN/Guangdong	2007.11	-	HQ659699			1	1	1
3	GZ08	CHN/Guangdong	2008.2.1	-	GQ478341			1	1	1
4	human/USA/CL_44/1988/7[P7H7F7]	USA	1988	7a	KF268125			2	2	2
5	NHRC 1315	USA	1997	7b	AY601634			2	2	2
6	human/USA/ak39_AdV7d2/1997/7[P7H7F7]	USA	1997	7d2	JX423387			2	2	2
7	human/USA/ak35_AdV7d2/2006/7[P7H7F7]	USA	2006	7d2	JX423383			2	2	2
8	FS2154	USA	2009	7d2	JN860677			2	2	2
9	hAdV/Saint-Petersburg/43/2013	RUS	2013.9.16	-	MG923582			2	2	2
10	19BOVLB/Volgograd/Rus/2014	RUS	2014.3.19	-	KU361344			2	2	2
11	CDC2014012.949	USA	2014.2.19	7d	KT963081			2	2	2
12	HAdV-B/USA NJ/5649/2016/ P7H7F7	USA	2016.12.19	7d	MH262321			2	2	2
13	HAdV-B/USA VA/5682/2017/ P7H7F7	USA	2017.8.3	7d	MH697601			2	2	2
14	HAdV-B/USA NJ/6296/2017/ P7H7F7	USA	2017.2.9	-	MH262324			2	2	2
15	0901HZ/Shx/CHN/2009	CHN/Shaanxi	2009.1.8	-	JF800905			2	2	2
16	Human/CHN/GZ6080/2010/7P7H7F7	CHN/Guangdong	2010.7.20	7d	KP670858			2	2	2
17	CQ1198	CHN/Chongqing	2010.11	7d	JX625134			2	2	2
18	Human/CHN/DG01/2011/7P7H7F7	CHN/Guangdong	2011	7d	KC440171			2	2	2
19	Human/CHN/GZ6965/2011/7P7H7F7	CHN/Guangdong	2011.8.8	7d	KP670856			2	2	2
20	HAdV B7 XZ2011-93	CHN/Jiangsu	2012.1.25	-	KC857700			2	2	2
21	CDC228/JM/CHN/2012	CHN/Hubei	2012	-	KJ019884			2	2	2
22	Human/CHN/GZ8546/2012/7P7H7F7	CHN/Guangdong	2012.4.26	-	KP670860			2	2	2
23	XY6/XY/CHN/2013	CHN/Hubei	2013	-	KJ019879			2	2	2
24	Shanxi/TY01/2013	CHN/Shanxi	2013.1.19	-	KP896480			2	2	2
25	Z9/WH/CHN/2015	CHN/Hubei	2015	-	KX897164			2	2	2
26	X07s	CHN/Jiangxi	2017.4.28	-	MG736303			2	2	2
27	X10s	CHN/Jiangxi	2017.4.30	-	MG736304			2	2	2
28	BJ/CHN/2018	CHN/Beijing	2018.1.1	-	MH355567			2	2	2
29	BJ01/CHN/2011	CHN/Beijing	2011	-	KP270906	KM458622	KP270915	2	2	2
30	BJ07/CHN/2013	CHN/Beijing	2013	-	KP270910	KM458626	KP270919	2	2	2
31	S-1058	USA	1955	7a	-	AF065066	AY921616	-	2	2
32	55142	USA	1963	7a	-	AF065067	-	-	2	-
33	Bal	JPN	1995	7d2	-	AF053087	AY921620	-	2	2
34	95–81	KOR	1995.10	7d	-	AY769945	AY921621	-	2	2
35	Kn T96-0620	USA	1996	7a	-	AF065068	-	-	2	-
36	99–95	KOR	1999.1	7l	-	AY769946	AY921617	-	2	2
37	TW-05159-2002	TWN	2002.10.11	-	-	KX570681	-	-	2	-
38	TW-02714-2004	TWN	2004.10.13	-	-	KX570679	-	-	2	-
39	0864/Taiwan/2011	TWN	2011	-	-	KC456143	KC456140	-	2	2
40	GZ22	CHN/Guangdong	2010	-	-	KJ195466	KJ195467	-	2	2
41	1106/SJZ/CHN/2011	CHN/Hebei	2011	-	-	JQ360622	JQ410443	-	2	2
42	SXWN1204	CHN/Shaanxi	2012.2.26	-	-	KC689914	-	-	2	-
43	383	JPN	1992	7d	-	-	AY921618	-	-	2
44	98–330	KOR	1998.7	7d	-	-	AY921622	-	-	2
45	AF2	KOR	2005	-	-	-	GQ265865	-	-	2
46	AF3	KOR	2005	-	-	-	GQ265866	-	-	2
47	DA4	KOR	2006	-	-	-	GQ265867	-	-	2
48	DA10	KOR	2006	-	-	-	GQ265873	-	-	2
49	SLE/2008/USA	USA	2008	7d2	-	-	HM057190	-	-	2
50	VM	USA	2014.12	7d	-	-	KT266800	-	-	2
51	Vaccine strain	USA	1950s	-	AY594256			3	2	2
52	HAdV-B/USA/55142/1960s	USA	1963	-	MH910669			3	2	2
53	human/USA/CL_43/1988/7[P7H7F7]	USA	1988	-	KF268134			3	2	2

### Bioinformatics analysis

Multiple sequence alignment and phylogenetic analysis within each dataset were conducted using MEGA version 6.0. To ensure the accuracy of the results, both maximum likelihood (ML) and neighbor-joining (NJ) phylogenetic trees were generated and tested by the bootstrap method with 1000 replications. Bootstrap values greater than 80% were considered strong support for the grouping. The similarity between sequences within each gene was evaluated using BioEdit version 7.1.7. The evolutionary rate for HAdV-7 was estimated with a coalescent-based Bayesian method implemented in BEAST version 1.7.4 (http://beast.community/).

### Nucleotide sequence accession numbers

The nucleotide sequences of the complete penton base, hexon, and fiber genes from 18 representative HAdV-7 strains in this study were submitted to the GenBank database with the following accession numbers MT019924-MT019941 (penton base), MT019906-MT019923 (hexon), and MT019942-MT019959 (fiber).

## Results

### Clinical features of HAdV-7 infection cases

Among the 122 HAdV-7-positive cases identified in this study, 29 (23.8%) were related to upper respiratory tract infections (URIs; median age: 18 years), whereas 93 (76.2%) were related to lower respiratory tract infections (median age: 2 years). Moreover, 27 (22.1%) cases were related to severe pneumonia (median age: 1.2 years). No deaths were reported. HAdV-7 infections occurred throughout the year, and most cases were detected in January (23%; Table 3).

**Table 3:** Clinical information of 122 HAdV-7-positive samples.

Disease	Clinical diagnosis	Specimen type	Month of collection/No. of Samples	Number of cases/Proportion	Age (median)
URI	URI, pharyngitis, tonsillitis	nasopharyngeal swab	1/1, 2/2, 3/14, 4/3, 5/1, 7/4, 9/3, 12/1	29	18
LRI	pneumonia asthmatic pneumonia, bronchopneumonia,	bronchoalveolar lavage fluid, sputum, Nasopharyngeal swab	1/25, 2/1, 3/4, 4/5, 5/8, 6/10, 7/5, 8/1, 10/1, 11/4, 12/2	66	2
	severe pneumonia	bronchoalveolar lavage fluid	1/2, 2/4, 3/3, 4/4, 5/5, 6/2, 7/3, 9/1, 11/1, 12/2	27	1.2

### Subtype division of HAdV-7 based on three capsid genes

**Fig 1:**
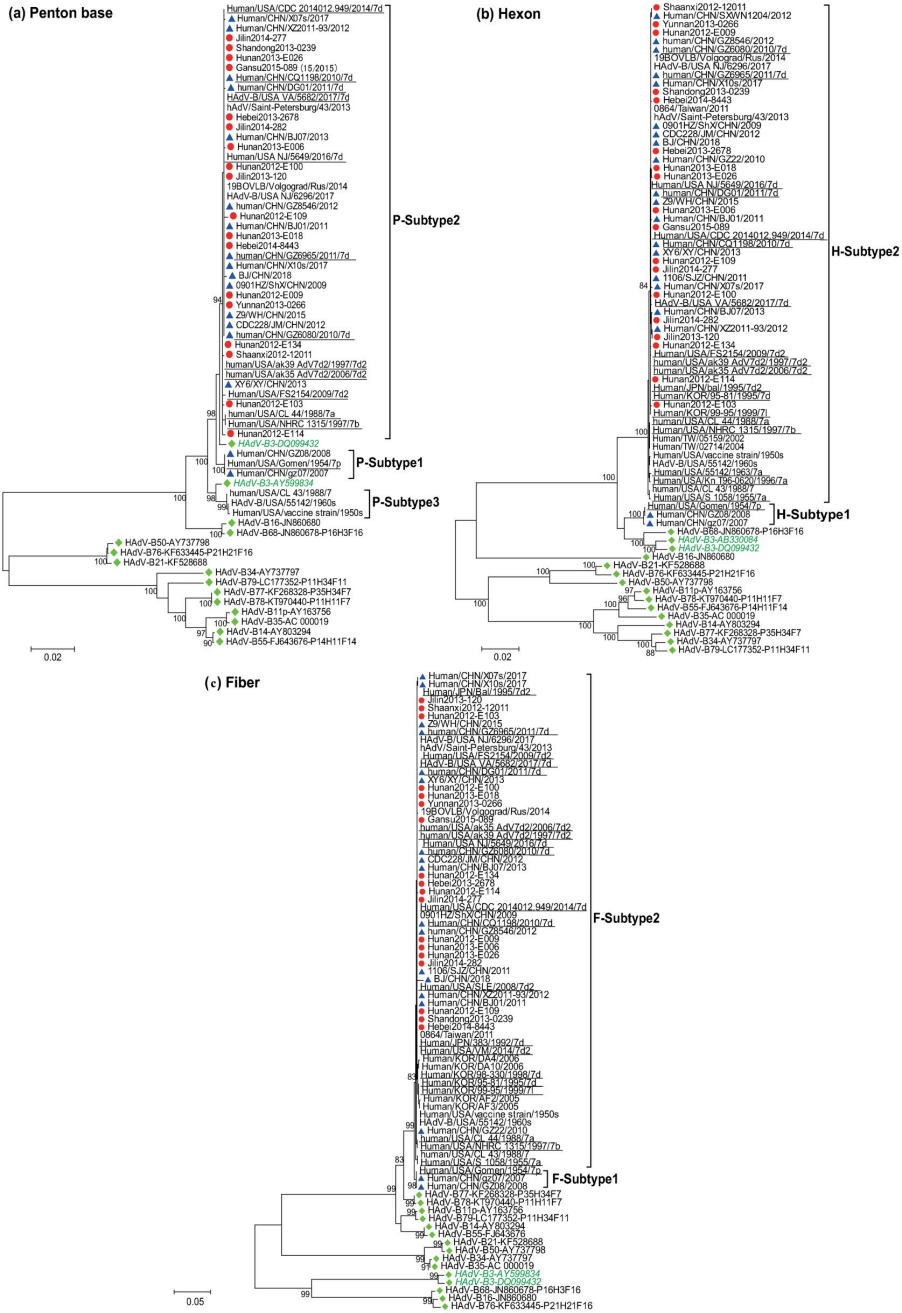
Neighbor-joining phylogenetic tree of HAdV-7 sequences based on penton base (a), hexon (b), and fiber genes (c). The red circles indicate the HAdV-7 strains identified in this study; blue triangles indicate the HAdV-7 strains isolated in China from GenBank database; underlined strains are known types of HAdV-7 identified by REA; green diamonds indicate other members within species B.

For genetic analysis of 69 HAdV-7 strains isolated during 2012–2015 in this study, the complete sequences of penton base, hexon, and fiber genes were amplified by PCR. The sequences of the three genes were similar among different strains (< 0.4% difference) or identical; 18 HAdV-7 strains were selected as representative viruses for further analysis (Table 1). Phylogenetic trees were constructed based on the established datasets of the three genes. The results showed that the topological structures of NJ trees were consistent with those of ML trees. Thus, only the NJ tree was presented (Fig 1).

With strong support of bootstrap values (> 80%), both hexon and fiber trees indicated that all sequences could be divided into two lineages, designated subtype 1 and subtype 2. Three strains, including HAdV-7 prototype stains from 1954 (strain Gomen) and two strains isolated in China from 2007 and 2008 (strains gz07 and GZ08), were grouped as subtype 1. All other 31 international HAdV-7 strains from five countries and regions isolated during 1955–2017 and 25 HAdV-7 strains (19 from GenBank database and six from this study) covering 14 provinces of China circulating since 2009 formed a separate main lineage of HAdV-7, which was recognized as subtype 2 (Fig 1). The genetic distances between two subtypes were 3.4% for hexon and 0.8% for fiber, respectively (Table 4). The results indicated that subtype 2 was the major type circulating worldwide since 1955.

**Table 4:** Average genetic distance within and between subtypes based on penton base, hexon, and fiber gene.

Gene	Subtype	Number of analyzed sequences	Average genetic distance	
			Within subtype	Between subtypes
Penton base	P-Subtype1	3	0.000	P-Subtype1/2: 0.007
	P-Subtype 2	45	0.001	P-Subtype1/3: 0.017
	P-Subtype 3	3	0.001	P-Subtype2/3: 0.017
Hexon	H-Subtype1	3	0.001	0.034
	H-Subtype 2	60	0.001	
Fiber	F-Subtype1	3	0.000	0.008
	F-Subtype 2	63	0.001	

The 51 penton base sequences exhibited more heterogeneity than hexon and fiber genes and featured three genetic lineages with a bootstrap value of greater than 94%. An additional genetic group (subtype 3) including the HAdV-7 vaccine strain from USA and two USA strains isolated in the 1960s and in 1988, was also identified (Fig 1). The genetic distances between the three subtypes were 0.7% (subtypes 1 and 2) and 1.7% (subtypes 2 and 3, subtypes 1 and 3; Table 4). All mean genetic distances within subtypes of penton base, hexon, and fiber genes were less than 0.1%, indicating that the three gene sequences in HAdV-7 were well conserved across the subtypes, but with relatively higher divergence between subtypes.

Compared with other members within species B, phylogenetic analysis of penton base and hexon genes indicated that HAdV-7 had a close genetic relationship with HAdV-3 (Fig 1). For the penton base gene, because the HAdV-3 strain isolated in China in 2005 (strain Guangzhou01, DQ099432) and the HAdV-3 prototype strain isolated in 1954 (strain GB, AY599834) were closer to subtypes 2 and 3, respectively, the genetic distance between them was less than 0.8%. For the hexon gene, both HAdV-3 strains were closer to subtype 1, and the genetic distance between them was 3.4%. This result indicated that HAdV-3 and HAdV-7 may have originated from a common ancestor.

### Sequence variations among HAdV-7 subtypes

Based on the three capsid genes evaluated in this study, the nucleotide and amino acid similarities of all HAdV-7 sequences were 98.1–100% and 97.9–100%, respectively, for penton base, 96.1–100% and 96.9–100%, respectively, for hexon, and 98.2–100% and 96.6–100%, respectively, for fiber. Further analysis of the sequences within the subtypes showed that only 3-bp synonymous mutations in the hexon gene were found for subtype 1. Additionally, nucleotide variations in subtype 3 sequences in the penton base gene were very low (< 0.2%). Similar results were observed for subtype 2; the minimum nucleotide and amino acid sequence identities for the three genes were 98.9% and 98.1%, respectively, except for one strain (BJ/CHN/2018) from China, which exhibited relatively higher variation (nine unique variations) in the fiber gene. The results further confirmed that the sequences within the subtypes of HAdV-7 were highly conserved.

**Fig 2:**
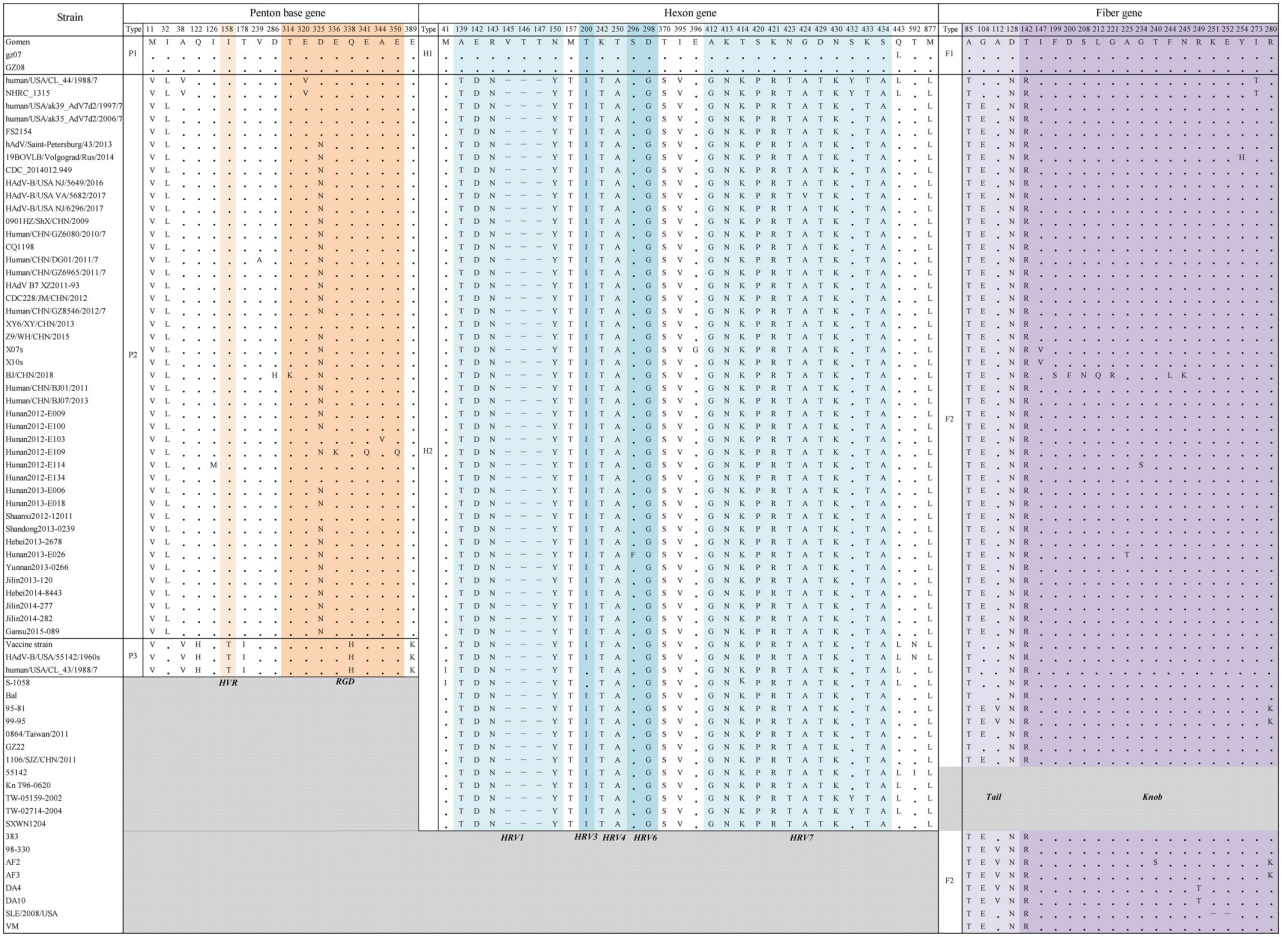
Amino acid variations in HAdV-7 strains based on three target genes. Dots represent sequences consistent with the HAdV-7 prototype strain (Gomen); horizontal lines represent base deletions; shadows represent virus strains lacking the relevant gene sequences.

Further analysis of the amino acid sequences of the three genes showed that the variations were closely related to the different subtypes and that viruses within the same subtype shared similar variation sites. These variation sites were mainly concentrated in important functional domains and hypervariable regions. In addition, there were no substantial differences between sequences according to the severity of HAdV-7 infection in this study (Fig 2).

Since 1978, at least 19 subtypes of HAdV-7 had been identified based on restriction enzyme analysis (REA) [16]. Compared with sequences for known genotypes listed in Table 2, no obvious characteristic differences were observed among different types across three genes, e.g., strain human/USA/CL_44/1988/7[P7H7F7] (genotype 7a), strain NHRC 1315 (genotype 7b), strain hAdV/Saint-Petersburg/43/2013 (genotype 7d2), and strain CQ1198 (genotype 7d; Fig 1).

### Evolutionary analysis of HAdV-7 subtype 2

Because subtype 2 was the predominant subtype worldwide since the 1950s, its evolutionary rate was estimated based on three genes. We detected different evolutionary rates for different genes. Penton base (9.52 × 10^−5^ substitutions/site/year; 95% HPD: 2.79 × 10^−5^–1.71 × 10^−4^) had a faster evolutionary rate than hexon (2.27 × 10^−5^ substitutions/site/year; 95% HPD: 9.80 × 10^−6^–3.66 × 10^−5^) and fiber genes (7.58 × 10^−5^ substitutions/site/year; 95% HPD: 3.34 × 10^−5^–1.25 × 10^−4^).

## Discussion

HAdV-7 is prevalent worldwide and accounts for approximately 20% of all adenoviral respiratory infections [17, 18]. In China, reports on HAdV-7 infections were found as early as the 1950s [19]. During the 1950s to 1970s, a serious epidemic of HAdV-associated infantile pneumonia occurred in northern China, and the fatality rate associated with HAdV-7 was up to 24% [19]. Since then, because of the absence of a national surveillance system, few reports of HAdV-7-related acute respiratory disease (ARD) have been published until recently. However, HAdV-7 epidemics have been frequent reported in western China (Shaanxi province in 2009 [18] and 2012 [11], Chongqing city during 2009–2012 [20]), central China (Hubei in 2012 and 2013) [21], and eastern China (Guangzhou city in 2011 [22], Beijing in 2012–2013 [21], Zhejiang and Shanghai in 2014 [12]). In particular, clusters of severe and fatal cases have occurred during HAdV-7 epidemics, making this virus a major public health threat.

Through the FRS system during 2012–2015, 122 HAdV-7 samples covering seven more provinces were identified from the ARD cases in the current study. Consistent with the epidemic patterns reported in previous studies [8, 23], HAdV-7 infection occurred throughout the year, and a high proportion of severe cases (22.1%) was found among pediatric patients, particularly those less than 2 years of age (mean age: 14 months). The clinical symptoms caused by HAdV-7 are not specific; thus, timely pathogenic screening is essential for early treatment of severe cases and to further reduce mortality rates.

To explore the genomic variability of HAdV-7, REA is commonly used for genotyping worldwide [16]. However, because of identification of new recombinant HAdVs (HAdV53–103) in recent years, genotyping based on only a few restriction sites can lead to misclassification of the virus strain. Therefore, as recommended by an international HAdV working group, the sequences of at least three target genes, including penton base, hexon, and fiber genes, should be used for preliminary genotyping, and determination of the whole genome sequence is also necessary if a potential new recombinant virus is identified (http://hadvwg.gmu.edu/).

In the current study, phylogenetic analysis of HAdV-7 sequences based on three major capsid genes showed that all HAdV-7 strains could be divided into two subtypes (subtypes 1 and 2), as reported previously [24]. Subtype 1 has been reported fewer times than subtype 2 worldwide. Limited data have shown that subtype 1 virus can be traced back to 1954, when the HAdV-7 prototype strain (Gomen, HAdV-7p) was detected in the USA; this strain became the major virus type circulating in the USA during 1966–2000 [17]. In China, an HAdV-7 prototype-like strain was identified in Beijing in 1981 [19] and in Guangzhou in 2007 and 2008. However, no subtype 1 viruses have been reported since 2008.

Subtype 2 virus, including some known types of HAdV-7 identified by REA (e.g., HAdV-7a, -7b, -7d2, -7d, and -7l), was also first detected in the 1950s. In contrast to subtype 1, subtype 2 viruses have been greatly expanding worldwide since the 1980s [2, 4, 14, 25–27], and this subtype has gradually became the predominant subtype worldwide, causing substantial increases in mortality and morbidity in recent years. In China, a long-term survey of adenoviral-related pneumonia since 1958 indicated that HAdV-7b was the predominant type before 1980; HAdV-7d then became the predominant type from 1980 to approximately 1990 [19, 28]. Moreover, after nearly two decades without detection, HAdV-7d has re-emerged, inducing high severity illness [22, 29]. Notably, all strains covering 14 provinces of China isolated after 2008 in the dataset in the current study belonged to subtype 2. The results indicated that the two subtypes of HAdV-7 cocirculated in China before 2008, with subtype 2 virus being the predominant subtype in recent years [22, 29]. However, because of the limited surveillance data, it is impossible to explain the reason for this subtype replacement.

We also identified subtype 3 virus from a vaccine (1950s) and two viruses (1960s and 1988) isolated in the USA based on the penton gene and supported by a high bootstrap value. Further amino acid sequence analysis of the three marker genes evaluated in this study indicated that all HAdV-7 viruses shared similar variation sites within subtypes for the three genes, further confirming the evolutionary independence of the subtypes. The nucleotide diversity of the penton base gene was also found for HAdV-C viruses [30].

Homologous recombination is an important feature for molecular evolution and immune escape of HAdV [31]. However, the results of this study indicated that the three major capsid gene of HAdV-7 were relatively stable across time and geographic space, particularly for viruses within the same subtype. For example, only 3 bp synonymous mutations were found for subtype 1 viruses across a 54-year interval, and subtype 2 viruses had relatively low evolutionary rates (2.3 × 10^−5^–9.5 × 10^−5^) and low mutation rates in important functional domains across a 50-year interval. This feature was also found for other types of HAdVs, such as HAdV-3 [32]. This lack of variability may be related to the strong proofreading ability of HAdV DNA polymerase, which greatly improves the fidelity of genome replication [32]. The relative stable genome of the virus could maintain the long-term effectiveness of the vaccine, given the fact that the HAdV-7 vaccine (subtype 3 of penton base genes and subtype 2 of hexon and fiber genes) has been administered to members of the USA military since the 1970s. This vaccine greatly reduced the incidence of ARDs caused by HAdV-7 in military camps [33]. Therefore, the development of HAdV-7 vaccines in China is expected to be feasible.

In this study, we were interested in one strain (BJ/CHN/2018) isolated from China in 2018. We found that this strain had nine unique variations in the fiber knob region, which has receptor-binding activity and thus mediates primary interactions with host cells, including cell tropism. Variations in this region may affect the pathogenicity of the virus [34], as was demonstrated in strains isolated in a Korean study in 1995–2000; amino acid changes in the receptor-binding domain of the fiber gene and E4 ORF 6/7 region were detected in HAdV-7l spread throughout Korea after 1996, allowing this virus to become the predominant strain in 1998–2000 [13]. Therefore, continuous surveillance of HAdV-7 is urgently required.

In recent years, outbreaks of respiratory tract infections caused by HAdV have occurred frequently in China, and clusters of severe or fatal cases have appeared in some areas [18, 29, 35]. To guide medical institutions and local CDCs in the detection, reporting, investigation, and elimination of outbreaks of adenoviral respiratory infections, China issued a document, titled “Technical guidelines for the prevention and control of human adenovirus respiratory tract infection”, in August 2019. Based on our findings, we suggest that a national HAdV surveillance system should be established as soon as possible to better define epidemic patterns of adenoviral respiratory infections, monitor HAdV outbreaks in real time in China, and clarify the genetic characteristics of HAdVs currently circulating in China. Such an approach would facilitate the development of effective preventive and infection-control interventions for adenovirus infection.

## Supporting information

S1 Table Clinical information for 122 HAdV-7-positive cases. (PDF)
