# Correction: Genetic variability of human adenovirus type 7 circulating in mainland China

**DOI:** 10.1371/journal.pone.0234681

**Published:** 2020-06-09

**Authors:** Ru Cai, Naiying Mao, Jingjing Dai, Xingyu Xiang, Jing Xu, Yingwei Ma, Zhong Li, Guangyue Han, Deshan Yu, Jie Yin, Aili Cui, Yan Zhang, Hong Li, Pengbo Yu, Luyuan Guan, Yuling Tian, Liwei Sun, Yan Li, Yamei Wei, Zhen Zhu, Wenbo Xu

There is an error in affiliation 2 for authors Naiying Mao, Aili Cui, Yan Zhang, Zhen Zhu, and Wenbo Xu. The correct affiliation 2 is: NHC Key Laboratory of Medical Virology and Viral Diseases, National Institute for Viral Disease Control and Prevention, Chinese Center for Disease Control and Prevention, Beijing, People’s Republic of China.
