# Supplementary material for: Genetic variability of human adenovirus type 7 circulating in mainland China
Source: PLoS One. 2020 Apr 30;15(4):e0232092. doi: 10.1371/journal.pone.0232092 (PMC7192419; doi:10.1371/journal.pone.0232092)
Supplement: S1 Table — (PDF) [file pone.0232092.s001.pdf]

Supplementary Table1. The information of 122 HAdV-7 positive cases

No.	Strain Name	Gender	Age	Date of Onset	Specimen	Clinical diagnosis	Case Type	Virus strain obtained
1	Shaanxi2012-12054	Male	19	2012/3	Nasopharyngeal swab	Upper respiratory tract infection	Outpatient	√
2	Shaanxi2012-12011	Male	18	2012/3	Nasopharyngeal swab	Upper respiratory tract infection	Outpatient	√
3	Shaanxi2012-12039	Male	18	2012/3	Nasopharyngeal swab	Upper respiratory tract infection	Outpatient	
4	Shaanxi2012-12026	Male	18	2012/3	Nasopharyngeal swab	Upper respiratory tract infection	Outpatient	
5	Shaanxi2012-12027	Male	18	2012/3	Nasopharyngeal swab	Upper respiratory tract infection	Outpatient	√
6	Shaanxi2012-12022	Male	18	2012/3	Nasopharyngeal swab	Upper respiratory tract infection	Outpatient	√
7	Shaanxi2012-12020	Male	18	2012/3	Nasopharyngeal swab	Upper respiratory tract infection	Outpatient	√
8	Shaanxi2013-13109	Male	9	2013/9	Nasopharyngeal swab	Upper respiratory tract infection	Outpatient	√
9	Shaanxi2012-12048	Male	17	2012/3	Nasopharyngeal swab	Upper respiratory tract infection	Outpatient	
10	Shaanxi2012-12046	Male	18	2012/3	Nasopharyngeal swab	Upper respiratory tract infection	Outpatient	
11	Shaanxi2012-12016	Male	18	2012/3	Nasopharyngeal swab	Upper respiratory tract infection	Outpatient	√
12	Shaanxi2012-12050	Male	23	2012/3	Nasopharyngeal swab	Upper respiratory tract infection	Outpatient	
13	Shaanxi2012-12024	Male	18	2012/3	Nasopharyngeal swab	Upper respiratory tract infection	Outpatient	
14	Shaanxi2013-13108	Female	10	2013/9	Nasopharyngeal swab	Upper respiratory tract infection	Outpatient	
15	Shaanxi2012-12023	Male	18	2012/3	Nasopharyngeal swab	Upper respiratory tract infection	Outpatient	
16	Shaanxi2012-12019	Male	18	2012/3	Nasopharyngeal swab	Upper respiratory tract infection	Outpatient	√
17	Hunan2014-106	Male	2	2014/1	Bronchoalveolar lavage fluid	Bronchopneumonia	Inpatient	
18	Hunan2014-123	Male	4	2014/1	Bronchoalveolar lavage fluid	Bronchopneumonia	Inpatient	√
19	Hunan2014-012	Female	6m	2014/1	Bronchoalveolar lavage fluid	Asthmatic pneumonia	Inpatient	
20	Hunan2014-015	Male	2	2014/2	Bronchoalveolar	Bronchopneumonia	Inpatient	

					lavage fluid			
21	Hunan2014-016	Male	14m	2014/1	Bronchoalveolar lavage fluid	Bronchopneumonia	Inpatient	√
22	Hunan2014-030	Male	3	2014/3	Sputum	Asthmatic pneumonia	Inpatient	
23	Hunan2014-034	Male	18m	2014/3	Bronchoalveolar lavage fluid	Severe pneumonia	Inpatient	√
24	Hunan2014-045	Male	13m	2014/3	Bronchoalveolar lavage fluid	Bronchopneumonia	Inpatient	√
25	Hunan2014-051	Female	3	2014/4	Bronchoalveolar lavage fluid	Right upper lung pneumonia	Inpatient	√
26	Hunan2014-058	Female	12m	2014/4	Bronchoalveolar lavage fluid	Bronchopneumonia	Inpatient	√
27	Hunan2014-060	Male	4	2014/4	Bronchoalveolar lavage fluid	Bronchopneumonia	Inpatient	√
28	Hunan2014-062	Male	17m	2014/4	Bronchoalveolar lavage fluid	Bronchopneumonia	Inpatient	√
29	Hunan2014-066	Female	3	2014/5	Bronchoalveolar lavage fluid	Upper left lung pneumonia	Inpatient	
30	Hunan2014-077	Male	2	2014/5	Bronchoalveolar lavage fluid	Lobar pneumonia of both lungs	Inpatient	
31	Hunan2014-082	Male	4	2014/6	Bronchoalveolar lavage fluid	Bronchopneumonia	Inpatient	
32	Hunan2014-084	Female	11m	2014/6	Bronchoalveolar lavage fluid	Double lung pneumonia	Inpatient	
33	Hunan2014-088	Male	3	2014/6	Bronchoalveolar lavage fluid	Bronchopneumonia	Inpatient	
34	Hunan2014-095	Male	3	2014/6	Bronchoalveolar lavage fluid	Bronchopneumonia	Inpatient	
35	Hunan2014-097	Female	16m	2014/6	Bronchoalveolar lavage fluid	Severe pneumonia	Inpatient	
36	Hunan2014-100	Male	5	2014/6	Bronchoalveolar lavage fluid	Bronchopneumonia	Inpatient	
37	Hunan2013-003	Male	2	2013/1	Bronchoalveolar lavage fluid	Bronchopneumonia	Inpatient	√
38	Hunan2012-004	Male	4	2012/12	Bronchoalveolar lavage fluid	Severe pneumonia	Inpatient	√
39	Hunan2013-006	Male	6	2013/1	Bronchoalveolar lavage fluid	Bronchopneumonia	Inpatient	√
40	Hunan2013-018	Male	6m	2013/1	Bronchoalveolar lavage fluid	Bronchopneumonia	Inpatient	√
41	Hunan2013-019	Female	2	2013/1	Bronchoalveolar lavage fluid	Bronchopneumonia	Inpatient	

42	Hunan2013-021	Female	Newborn	2013/2	Bronchoalveolar lavage fluid	Severe pneumonia	Inpatient	√
43	Hunan2013-022	Male	19m	2013/2	Bronchoalveolar lavage fluid	Severe pneumonia	Inpatient	√
44	Hunan2013-026	Male	18m	2013/2	Bronchoalveolar lavage fluid	Severe pneumonia	Inpatient	√
45	Hunan2013-047	Female	13m	2013/3	Bronchoalveolar lavage fluid	Severe pneumonia	Inpatient	
46	Hunan2013-052	Male	2	2013/4	Bronchoalveolar lavage fluid	Severe pneumonia	Inpatient	
47	Hunan2013-096	Male	14m	2013/7	Bronchoalveolar lavage fluid	Severe pneumonia	Inpatient	√
48	Hunan2013-109	Male	6m	2013/8	Bronchoalveolar lavage fluid	Bronchopneumonia	Inpatient	
49	Hunan2013-122	Female	3	2013/9	Bronchoalveolar lavage fluid	Severe pneumonia	Inpatient	
50	Hunan2013-145	Female	3	2013/11	Bronchoalveolar lavage fluid	Severe pneumonia	Inpatient	
51	Hunan2012-009	Male	16m	2012/1	Bronchoalveolar lavage fluid	Severe pneumonia	Inpatient	√
52	Hunan2012-027	Male	3m	2012/1	Bronchoalveolar lavage fluid	Severe pneumonia	Inpatient	√
53	Hunan2012-028	Female	2	2012/2	Bronchoalveolar lavage fluid	Severe pneumonia	Inpatient	√
54	Hunan2012-067	Male	13m	2012/3	Bronchoalveolar lavage fluid	Severe pneumonia	Inpatient	√
55	Hunan2012-093	Male	4	2012/4	Bronchoalveolar lavage fluid	Severe pneumonia	Inpatient	
56	Hunan2012-094	Male	3m	2012/4	Bronchoalveolar lavage fluid	Severe pneumonia	Inpatient	√
57	Hunan2012-100	Female	8m	2012/4	Bronchoalveolar lavage fluid	Severe pneumonia	Inpatient	√
58	Hunan2012-101	Male	2	2012/5	Bronchoalveolar lavage fluid	Bronchopneumonia	Inpatient	√
59	Hunan2012-103	Male	12m	2012/5	Bronchoalveolar lavage fluid	Severe pneumonia	Inpatient	√
60	Hunan2012-104	Male	8m	2012/5	Bronchoalveolar lavage fluid	Severe pneumonia	Inpatient	√
61	Hunan2012-105	Male	2	2012/5	Bronchoalveolar lavage fluid	Severe pneumonia	Inpatient	
62	Hunan2012-109	Male	6m	2012/5	Bronchoalveolar lavage fluid	Severe pneumonia	Inpatient	√
63	Hunan2012-110	Male	23m	2012/5	Bronchoalveolar lavage fluid	Severe pneumonia	Inpatient	

64	Hunan2012-111	Female	11m	2012/6	Bronchoalveolar lavage fluid	Bronchopneumonia	Inpatient	
65	Hunan2012-114	Male	6m	2012/6	Bronchoalveolar lavage fluid	Severe pneumonia	Inpatient	√
66	Hunan2012-123	Male	11m	2012/6	Bronchoalveolar lavage fluid	Bronchopneumonia	Inpatient	
67	Hunan2012-128	Male	3	2012/6	Bronchoalveolar lavage fluid	Left lower lung pneumonia	Inpatient	
68	Hunan2012-133	Female	19m	2012/7	Bronchoalveolar lavage fluid	Bronchopneumonia	Inpatient	√
69	Hunan2012-134	Male	3	2012/7	Bronchoalveolar lavage fluid	Bronchopneumonia	Inpatient	√
70	Hunan2012-140	Male	2	2012/7	Bronchoalveolar lavage fluid	Asthmatic pneumonia	Inpatient	
71	Hunan2012-145	Male	10m	2012/7	Bronchoalveolar lavage fluid	Severe pneumonia	Inpatient	√
72	Hunan2012-146	Male	4m	2012/7	Bronchoalveolar lavage fluid	Severe pneumonia	Inpatient	√
73	Hunan2012-193	Female	22m	2012/11	Bronchoalveolar lavage fluid	Bronchopneumonia	Inpatient	
74	Hunan2012-212	Male	11m	2012/12	Bronchoalveolar lavage fluid	Severe pneumonia	Inpatient	√
75	Hebei2012-9617	Male	53	2012/2	Nasopharyngeal swab	Pyogenic tonsillitis	Outpatient	
76	Hebei2012-0177	Male	18	2012/3	Nasopharyngeal swab	Pneumonia	Outpatient	
77	Hebei2014-4621	Male	12m	2014/1	Nasopharyngeal swab	Bronchopneumonia	Inpatient	
78	Hebei2013-2678	Male	23	2013/2	Nasopharyngeal swab	Upper respiratory tract infection	Outpatient	√
79	Hebei2014-8443	Male	25	2014/3	Nasopharyngeal swab	Pneumonia	Outpatient	√
80	Shandong2013-7627	Female	14	2013/4	Nasopharyngeal swab	Upper respiratory tract infection	Outpatient	√
81	Shandong2013-7585	Male	13	2013/4	Nasopharyngeal swab	Upper respiratory tract infection	Outpatient	√
82	Shandong2013-0239	Female	2	2013/1	Nasopharyngeal swab	Upper respiratory tract infection	Outpatient	√
83	Shandong2013-7569	Male	16	2013/4	Nasopharyngeal swab	Upper respiratory tract infection	Outpatient	
84	Shandong2014-8153	Female	7	2014/1	Nasopharyngeal swab	Pneumonia	Inpatient	√
85	Gansu2015-001	Female	2	2014/12	Nasopharyngeal swab	Bronchopneumonia	Inpatient	

86	Gansu2015-003	Male	7	2015/1	Nasopharyngeal swab	Bronchopneumonia	Inpatient	√
87	Gansu2015-007	Male	7	2015/1	Nasopharyngeal swab	Bronchopneumonia	Inpatient	√
88	Gansu2015-010	Male	7	2014/12	Nasopharyngeal swab	Bronchopneumonia	Inpatient	√
89	Gansu2015-012	Male	4	2015/1	Nasopharyngeal swab	Bronchopneumonia	Inpatient	√
90	Gansu2015-021	Male	5	2015/1	Nasopharyngeal swab	Bronchopneumonia	Inpatient	√
91	Gansu2015-051	Male	8	2015/1	Nasopharyngeal swab	Bronchopneumonia	Inpatient	√
92	Gansu2015-055	Female	6	2015/1	Nasopharyngeal swab	Bronchopneumonia	Inpatient	
93	Gansu2015-058	Female	12m	2015/1	Nasopharyngeal swab	Bronchopneumonia	Inpatient	√
94	Gansu2015-060	Male	10	2015/1	Nasopharyngeal swab	Bronchopneumonia	Inpatient	√
95	Gansu2015-063	Male	2	2015/1	Nasopharyngeal swab	Bronchopneumonia	Inpatient	√
96	Gansu2015-064	Male	2	2015/1	Nasopharyngeal swab	Bronchopneumonia	Inpatient	√
97	Gansu2015-075	Female	2	2015/1	Nasopharyngeal swab	Bronchopneumonia	Inpatient	√
98	Gansu2015-077	Male	11m	2015/1	Nasopharyngeal swab	Bronchopneumonia	Inpatient	√
99	Gansu2015-078	Female	4m	2015/1	Nasopharyngeal swab	Bronchopneumonia	Inpatient	√
100	Gansu2015-081	Male	6m	2015/1	Nasopharyngeal swab	Bronchopneumonia	Inpatient	√
101	Gansu2015-089	Female	4	2015/1	Nasopharyngeal swab	Bronchopneumonia	Inpatient	√
102	Yunnan2014-5324	Male	11	2014/9	Nasopharyngeal swab	Tonsillitis	Outpatient	
103	Yunnan2014-1831	Female	8	2014/12	Nasopharyngeal swab	Pharyngitis	Outpatient	
104	Yunnan2013-4038	Female	3	2013/5	Nasopharyngeal swab	Upper respiratory tract infection	Outpatient	√
105	Yunnan2013-8468	Female	4	2013/7	Nasopharyngeal swab	Pharyngitis	Outpatient	
106	Yunnan2013-8450	Female	9	2013/7	Nasopharyngeal swab	Pharyngitis	Outpatient	
107	Yunnan2013-8526	Male	8	2013/7	Nasopharyngeal swab	Pharyngitis	Outpatient	√

108	Yunnan2013-0266	Female	5	2013/7	Nasopharyngeal swab	Tonsillitis	Outpatient	√
109	Jilin2013-120	Female	4	2013/10	Nasopharyngeal swab	Bronchopneumonia	Inpatient	√
110	Jilin2013-191	Female	15m	2013/11	Nasopharyngeal swab	Bronchopneumonia	Inpatient	
111	Jilin2013-231	Female	4	2013/11	Nasopharyngeal swab	Bronchopneumonia	Inpatient	
112	Jilin2013-251	Male	4	2013/11	Nasopharyngeal swab	Bronchopneumonia	Inpatient	
113	Jilin2014-277	Female	4	2014/4	Nasopharyngeal swab	Bronchopneumonia	Inpatient	√
114	Jilin2014-282	Male	2	2014/5	Nasopharyngeal swab	Bronchopneumonia	Inpatient	√
115	Jilin2014-295	Female	8m	2014/5	Nasopharyngeal swab	Bronchopneumonia	Inpatient	
116	Jilin2015-296	Male	6m	2015/7	Nasopharyngeal swab	Bronchopneumonia	Inpatient	√
117	Jilin2014-301	Male	2	2014/6	Nasopharyngeal swab	Bronchopneumonia	Inpatient	
118	Jilin2014-302	Male	9m	2014/6	Nasopharyngeal swab	Bronchopneumonia	Inpatient	√
119	Jilin2014-304	Male	4m	2014/5	Nasopharyngeal swab	Bronchopneumonia	Inpatient	
120	Jilin2014-310	Female	4m	2014/5	Nasopharyngeal swab	Bronchopneumonia	Inpatient	
121	Jilin2014-316	Female	6	2014/5	Nasopharyngeal swab	Bronchopneumonia	Inpatient	
122	Jilin2014-366	Male	3	2014/7	Nasopharyngeal swab	Bronchopneumonia	Inpatient	
